# Research on the Mechanism of Oxygen-Induced Embrittlement Fracturing in Industrial Electrolytic Nickel

**DOI:** 10.3390/ma17174428

**Published:** 2024-09-09

**Authors:** Han Zhang, Chen Sang, Chengpeng Miao, Yangtao Xu, Jisen Qiao, Tiandong Xia

**Affiliations:** 1 School of Materials Science and Engineering, Lanzhou University of Technology, Lanzhou 730050, China; 18793138809@163.com (H.Z.); 19193103682@163.com (C.S.); lanzhouxuyt@163.com (Y.X.); 2 State Key Laboratory of Advanced Processing and Recycling of Non-Ferrous Metal, Lanzhou University of Technology, Lanzhou 730050, China; 3 Jinchuan Group Nickel Alloy Co., Ltd., Jinchang 737100, China; cp.m0628@163.com

**Keywords:** electrolytic nickel, hot rolling, pores, grain boundary cavitation, high-temperature ductility dip

## Abstract

In this study, severe cracking occurred during an investigation of the direct hot rolling of industrial electrolytic nickel plates. To determine the cause of hot-rolling cracking, the microstructure phase composition was analyzed through the utilization of various techniques, including optical microscopy, scanning electron microscopy, electron backscattering diffraction, transmission electron microscopy (TEM), energy-dispersive X-ray spectroscopy (EDS) and electron probe micro-analysis. The comparative microstructural analysis took place between specimens heat treated in atmospheric and vacuum environments. The characterization and analysis of the hot-rolled plates considered the crack microstructure and fracture morphology. It was shown that holes appeared along the large angular grain boundaries after annealing at 1100 °C for 8 h. Possible reason: In a high-temperature environment, the decomposition of residual additives in the electrolytic nickel releases oxidizing gases, which oxidizes the grain boundaries. The reaction with carbon diffused into the grain boundaries and produced carbon monoxide gas, which induced holes and severely reduced the grain boundary plasticity. The heat treatment time did not need to be very long for severe grain boundary degradation to occur. After severe cavitation, the electrolytic nickel was severely cracked at grain boundaries cracks due to a shear force, and brittle fractures occurred along grains with very low plasticity.

## 1. Introduction

High-purity nickel has properties such as a clean microstructure, excellent mechanical and electrical conductivities, and excellent corrosion resistance. It is widely used in sputtering targets, temperature sensors, battery collectors, battery terminals, resistance thermometers and deep-drawn products [1,2,3]. Nickel is initially produced in the form of cathode plates or powder, both of which are 99.98% pure. They are cut into squares as raw material; then electroplated and dissolved or directly melted; and finally used to produce pure nickel, nickel alloys or stainless steel.

The cathode nickel plate (electrolytic nickel) produced by electrolytic deposition is of high purity, and therefore, attempts are often made to process it directly into industrial nickel products by producing high-purity nickel products directly from the cathode plate. However, these attempts are not easily successful due to the presence of certain negative characteristics of cathode nickel plates that cannot be fully overcome. However, Theodor Stut et al. [4] investigated a new process for the preparation of high-purity nickel wires and developed a process for manufacturing high-purity nickel wires directly from cathode plates.

Cathode plates are produced by placing a thin starting sheet in an electrolytic bath and the production process involves the deposition of nickel ions from the electrolyte onto the nickel starting sheet, layer by layer. A layer of nickel is deposited on both sides of the starting sheet. As a result, the cathode plate consists of three layers [5,6] with significant layering. Cathode plates contain hydrogen [7], which can cause hydrogen embrittlement during processing. All of these problems mean that the plates are susceptible to cracking or shape problems during rolling.

The nucleation and growth processes of electrolytic nickel, which is produced through electrodeposition, is influenced by the composition of the electrolytic liquid system and the electrolytic techniques employed. Ultimately, this affects the quality and performance of electrolytic nickel. During the electrolytic process, the addition of additives, such as nucleating agents, brighteners, levelling agents and surfactants, can effectively improve the quality of the nickel deposit [8,9,10,11,12]. Xia et al. [13] compared the microstructure and mechanical properties of domestically produced electrolytic nickel sheets and rolled nickel sheets and found that electrolytic nickel sheets with a high purity, higher strength hardness and also good plastic toughness is suitable for the direct rolling of nickel strip foil. However, there are differences in the quality of domestic cathode nickel plate compared with foreign counterparts, where the domestic electrolytic nickel grain size has a large and uneven distribution and the mechanical properties have obvious directionality, among other problems. These problems also affect the direct roll formation.

Bricknell and Woodford [14] conducted a study on the tensile properties of Ni270. Nickel also suffers from along-grain fractures when samples are heated at very low oxygen partial pressures (<1.33 × 10^−5^ Pa) [15,16]. The penetration of atmospheric oxygen along grain boundaries at high temperatures is responsible for the embrittlement. Studies on high-purity nickel [17,18], commercially pure nickel [14,19,20], impurity-added nickel [21] and Ni (Bi) alloys [18] unequivocally demonstrated that ITE (intermediate-temperature embrittlement) is a consequence of impurities. Impurity levels as low as several parts per million are adequate to induce such embrittlement. In the event of the absence of carbon or sulfide in nickel, intergranular oxidation can also occur due to doping from trace impurities or intentionally added oxides [22].

During this study of the direct hot rolling of industrial electrolytic nickel, severe cracking occurred. The cold formability of electrolytic nickel is highly favorable. In prior research, it was demonstrated that the deformation of electrolytic nickel can achieve up to 98% during cold rolling processes without the need for intermediate annealing [5]. However, the cracking under high-temperature rolling conditions is significant. In order to determine the cause of hot-rolled cracking, a comparative analysis of the microstructures of specimens heat treated under atmospheric and vacuum conditions was carried out. A detailed analysis of the microstructure and fracture morphology of hot-rolled cracks was carried out. Identifying the embrittlement mechanism in the thermal deformation of industrial electrolytic nickel materials is of great significance in solving the problem of the high-temperature low plasticity of electrolytic nickel.

## 2. Materials and Experimental Methods

The electrolytic nickel used here consisted of nickel sulfide in sulfate solution, which was electrolytically refined and deposited bilaterally on a higher-purity nickel starting sheet. This resulted in a finished industrial electrolytic nickel product. Its main chemical composition is shown in Table 1.

**Figure 1:**
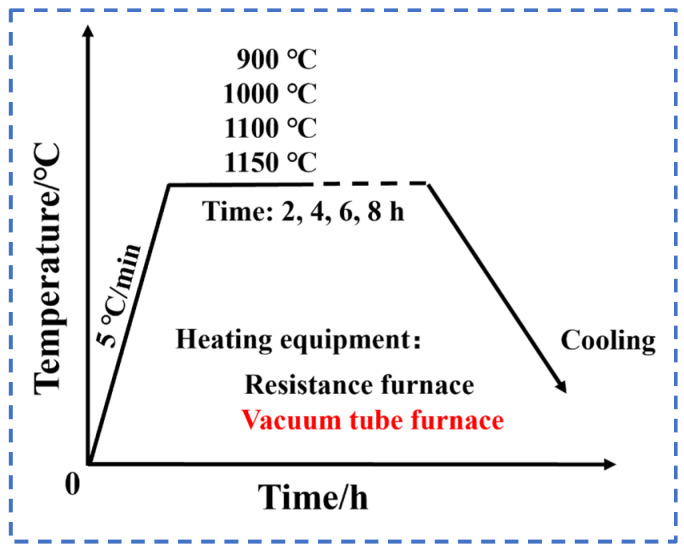
Schematic diagram of heat treatment process.

The specimens were machined on the electrolytic nickel plates using a wire-cutting machine, where the size of the heat-treated specimens was 100 mm × 20 mm × 10 mm. The heat treatment of the electrolytic nickel was carried out using an SX-G08163 muffle furnace (Tianjin Zhonghuan Electric Furnace Co., Tianjin, China) with different holding temperatures and times. The heat treatment scheme is shown schematically in Figure 1. The cooling method was air cooling. The comparative tests were carried out in a vacuum tube furnace. The sample was vacuum sealed in a glass quartz tube and then placed in the tube furnace to increase in temperature with the furnace. The holding temperature was 1100 °C and the holding time was 8 h. The cooling method was oven cooling.

**Figure 2:**
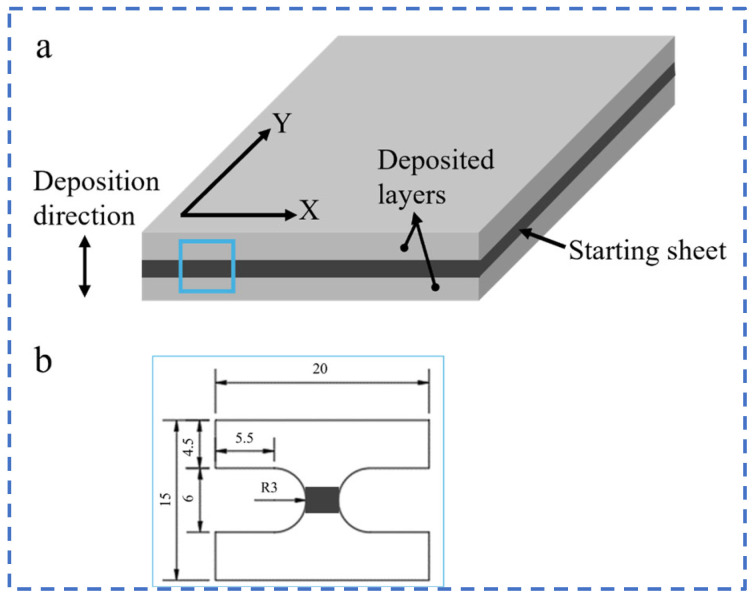
Schematic diagrams of electrolytic nickel and tensile test specimens: (**a**) position of the starting sheet and deposition layer; (**b**) tensile specimen size.

Figure 2 is a schematic diagram of an electrolytic nickel plate. The position of the nickel initiator sheet in the nickel plate is shown in Figure 2a, and the nickel deposition layer (deposition direction) was deposited on both sides of the initiator sheet. Due to the characteristics of the electrolytic nickel preparation process, tensile tests were carried out on the bonding properties between the initiator sheet and the deposited layer and between the deposited layers. The tensile test was performed using a WDW-100D electronic universal materials testing machine (Shandong Kaifeng Inspection Technology Co., Jinan, China) with a loading speed of 0.2 mm/s. Due to the small thickness of the electrolytic nickel in the deposition direction (18–23 mm), the tensile specimens were processed as non-standard specimens. The specimen area is shown in Figure 2a, the specimen size specification is shown in Figure 2b and the thickness of the tensile specimen was 1 mm.

Direct hot rolling of the electrolytic nickel plates was undertaken after edge trimming and surface grinding. The dimensions of the nickel plate were 800 mm × 660 mm, with thicknesses that ranged from 16 mm to 20 mm. The electrolytic nickel was subjected to a temperature of 1100 °C using a resistance-heating furnace for a duration of 8 h. Subsequently, it was removed for hot rolling, with the temperature maintained at approximately 1000 °C during this process.

Physical phase and crystal orientation tests were carried out on the samples using a RINT2000 X-ray diffractometer (XRD) (Nihon Rikaku Co., Tokyo, Japan) with the following parameters: a copper target, incident wavelength λ of 0.15406 nm, experimental tube voltage of 40 kV, tube current of 40 mA, scan range of 10° to 90° and scan step size of 0.02°. A Wilson 1102D37 fully automated microhardness tester (Buehler Ltd., Lake Bluff, IL, USA) was used for the hardness testing. The specimen size was 10 mm × 8 mm × 6 mm, which was machined by wire cutting to ensure parallelism of the top and bottom surfaces. The specimens were mechanically polished with sandpaper until the surface was smooth without obvious scratches. The test load was 0.2 kgf and the loading time was 12 s. Each specimen was tested at 30 points and the average value was taken as the hardness value.

The specimens were observed before and after the heat treatment by optical microscopy (OM) using an Axio Scope.A1 optical microscope (Carl Zeiss AG, Oberkochen, Germany). The specimens were gradually polished with sandpaper until the surface was smooth and without obvious scratches, and then mechanically polished to a mirror surface using a corrosive agent that contained distilled water: concentrated nitric acid: glacial acetic acid as 1:4:10.

The chemical composition of the inner grain boundary and the precipitated phase of the material was analyzed using the electron probe JXA-8530F PLUS (JEOL Co., Ltd., Tokyo, Japan). An Oxygen Nitrogen Hydrogen Analyzer ONH836 (LECO, Saint Joseph, MO, USA) was utilized for the analysis of the oxygen, nitrogen and hydrogen contents. The Carbon-Sulfur Analyzer HCS-140 (Shanghai Dekai Instrument Co., Shanghai, China) was utilized for the determination of the carbon and sulfur element contents. The Wavelength Dispersive X-ray Fluorescence Spectrometer Axios MAX (PANalytical B.V., Almelo, The Netherlands) was capable of detecting phosphorus.

The heat-treated tissues, hot-rolled cracked fractures and adjacent tissues were observed using a Quanta FEG-450 scanning electron microscope (SEM) (Oxford Instruments, Oxford, UK) and analyzed using energy dispersive spectroscopy (EDS) (Oxford Instruments, Oxford, UK). After sanding and electrolytic polishing, the microstructure at the cracks was observed using Oxford-SYMMETRY S3 electron backscatter diffraction (EBSD) (Oxford Instruments, Oxford, UK) with an electron acceleration voltage of 15 kV. The specimens were cut at the cracks using an EDM wire cutter (AVIC Changfeng CNC Technology Co., Suzhou, China), sanded to 30 μm and thinned using a Gatan ion thinning instrument (Gatan, Pleasanton, CA, USA) with an ion gun voltage of 6 kV. Microstructural characterization was carried out using an FEI Talos F200X transmission electron microscope (Thermo Fisher Scientific, Waltham, MA, USA) with an electron acceleration voltage of 200 kV.

## 3. Experimental Results and Analysis

### 3.1. Microstructure of Electrolytic Nickel

**Figure 3:**
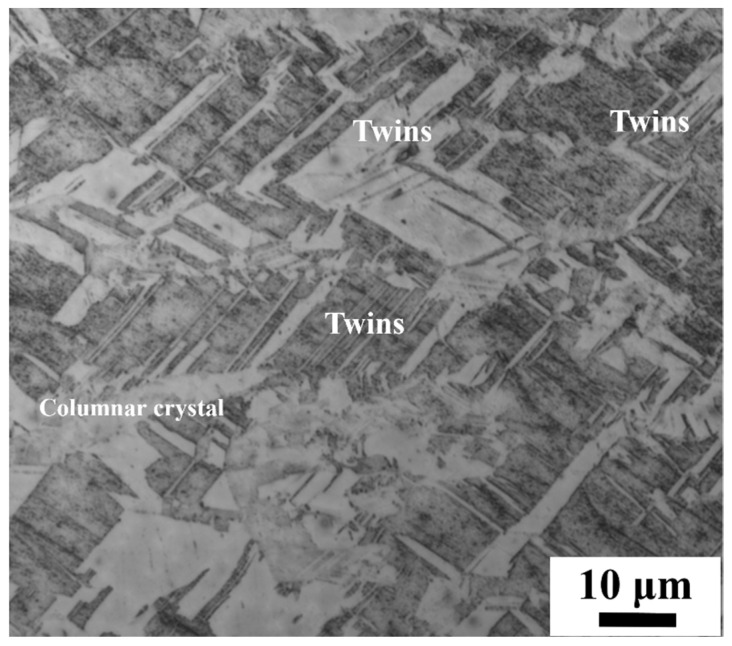
Microstructure of electrolytic nickel.

Figure 3 illustrates the microstructure of the raw material of electrolytic nickel. It can be seen that the electrolytic nickel grain size varied, with an average grain size of about 0.5 μm. The lamellar organization was growth twinning, which accounted for about 44%. Growth twins in industrial electrolytic nickel are not uniform in length and thickness. As the deposition process stabilized, columnar crystals began to grow and gradually took on their fast-growing form. Columnar crystals of various sizes that were interspersed with some fine equiaxed crystals can be seen in Figure 3.

### 3.2. Microstructure of Electrolytic Nickel after High-Temperature Heat Treatment

#### 3.2.1. Microstructure and Mechanical Properties of Electrolytic Nickel after Heat Treatment in Atmospheric Conditions

**Figure 4:**
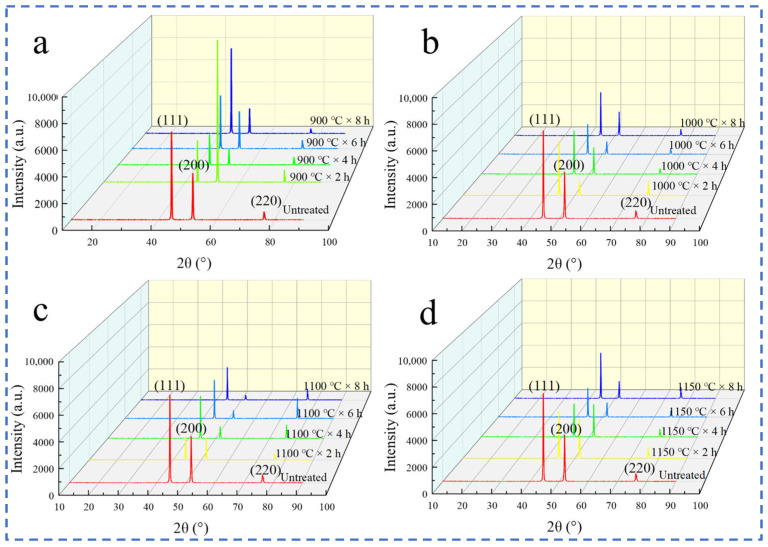
XRD patterns of electrolytic nickel after heat treatment: (**a**) 900 °C; (**b**) 1000 °C; (**c**) 1100 °C; (**d**) 1150 °C.

Figure 4 shows the XRD patterns of the electrolytic nickel heat treated at different holding temperatures and for different holding times. Figure 4a shows that the diffraction intensity of the (111) facet of the electrolytic nickel decreased and then increased with the increase in holding time, and it was the strongest diffraction intensity among the three grain facets. In the direction parallel to the deposition layer, the grains grew mainly perpendicular to the (111) facet. The (200) facet had an unusually high diffraction peak at 900 °C × 2 h. The diffraction intensity of the (220) facet decreased further. In Figure 4b, the diffraction intensities of the (111), (200) and (220) facets weakened rapidly with the heat treatment at 1000 °C, and the diffraction peak intensities gradually stabilized after 4 h. In Figure 4c, it was found that the diffraction intensities of the (111) and (220) facets decreased rapidly with the heat treatment at 1100 °C × 2 h and gradually stabilized after 4 h of heat treatment, and the intensity of the diffraction peaks of the (200) facet continued to decrease. In Figure 4d, the diffraction intensity of each facet was the lowest at 1150 °C × 6 h, and the overall trend was first weakened and then strengthened facets.

The general pattern before and after the heat treatment of electrolytic nickel was the presence of three crystal planes: the (111) plane, the (200) plane and the (220) plane. Among them, the diffraction peak of the (111) plane was always the stronger one among the three diffraction peaks. Electrolytic nickel belongs to the face-centered cubic structure, and its (111) face has the lowest surface energy. Therefore, electrolytic nickel grains grew along the densely packed (111) face, which had the highest diffraction peak intensity.

**Figure 5:**
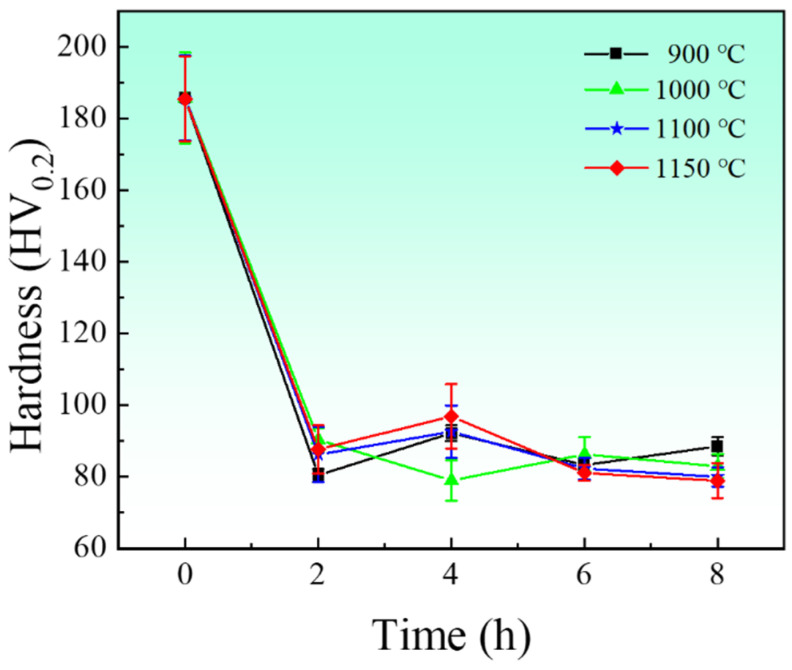
Average microhardness of electrolytic nickel after heat treatment.

Figure 5 shows the microhardness curve of the electrolytic nickel after the heat treatment. The hardness of the electrolytic nickel decreased rapidly with increased heat treatment temperature. It decreased from 185 HV to a minimum of 79 HV and a maximum of 57%. At high temperatures, the microscopic defects and microscopic strains of electrolytic nickel provide additional energy for the diffusion of nickel atoms. Microscopic defects, such as dislocations, disappear; internal stresses are gradually released; and grains gradually grow such that the hardness significantly decreases. Eventually, all hardnesses stabilized between 80 and 90 HV after 6 h of holding.

**Figure 6:**
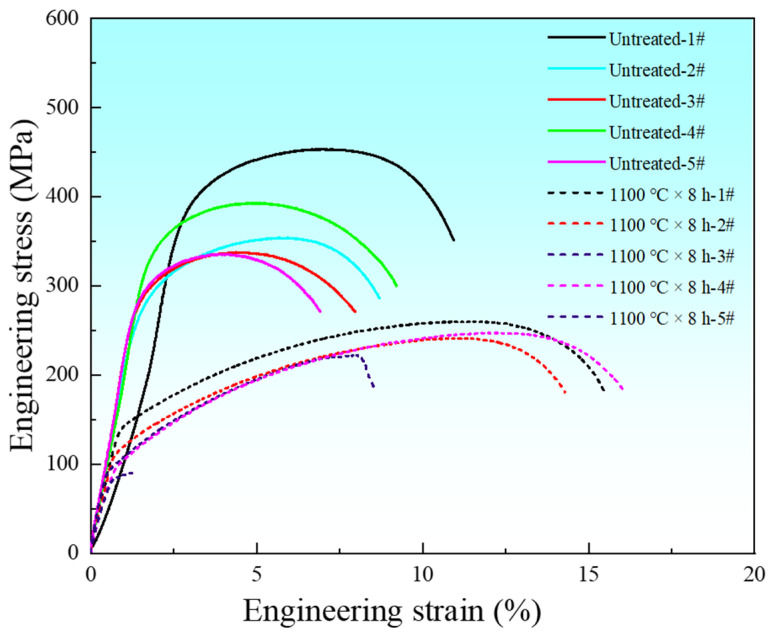
Stress–strain curves of electrolytic nickel tensile specimens before and after heat treatment.

Figure 6 shows the engineering stress–strain curves of the tensile specimens before and after the electrolytic nickel heat treatment. Five parallel specimens of the same specification were used for the experiment. The experimental results show that the performance of the electrolytic nickel varied greatly before and after the heat treatment. Before the heat treatment, the tensile strength of the electrolytic nickel could be as high as 455 MPa and as low as 136 MPa. The average tensile strength was 336.2 MPa. The elongation at break could be as high as 7.25% and as low as 2.78%. The average elongation at break was 5.04%. The tensile strength and elongation at break were relatively scattered.

After the heat treatment, the tensile strength of the electrolytic nickel was the highest at 260 MPa and the lowest at 109 MPa. The average tensile strength was 215.8 MPa and the elongation at break was the highest at 11.96% and the lowest at 5.13%. The average elongation at break was 9.59%. The mechanical properties in the direction of electrolytic nickel deposition before and after the heat treatment were unstable.

**Figure 7:**
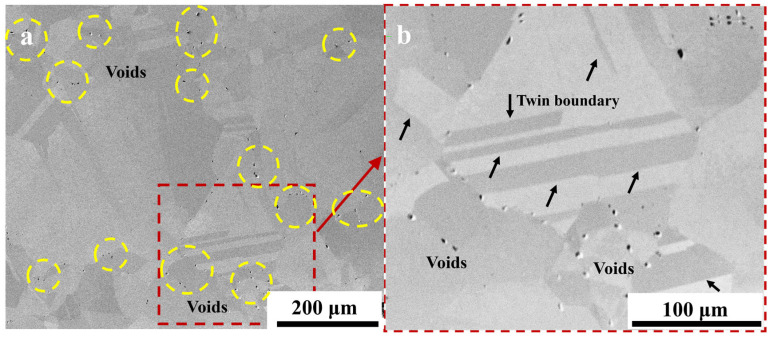
SEM microstructure of electrolytic nickel heat treatment (non-metallurgical corrosion): (**a**) microstructure following 8 h at 1100 °C in atmospheric condition; (**b**) voids along grain boundaries.

The microstructure of the electrolytic nickel after being subjected to an atmospheric heat treatment at 1100 °C for 8 h is illustrated in Figure 7. Under the elevated temperatures, there was an increase in the grain size of the material, which led to the disappearance of the original columnar crystal structure. Equiaxed crystals of various sizes were present, with an average size of approximately 100 μm. The original growth twins of the electrolytic nickel disappeared after the high-temperature treatment and a large number of annealed twins appeared. A significant quantity of annealed twins manifested in the microstructure following the heat treatment. In Figure 7, the annealed twins predominantly existed in the form of the penetrating grain type and non-penetrating grain type. These twins were generated through abrupt changes during the process of high-angle grain boundary (HAGB) migration in the high-temperature treatment. The primary factors that influenced the grain boundary migration were predominantly the temperature and energy gradients. The annealed twin boundary consistently initiated or terminated at a grain boundary. In face-centered cubic metals, the annealing twin boundary is the coherent twin boundary, which typically exhibits coherence with an orientation of (111). The twin boundary described exhibited a significantly low interfacial energy, which led to a reduction in the system’s overall energy upon the formation of such a twin boundary [23].

After the heat treatment, it is noteworthy that the voids were predominantly concentrated in the HAGB, with a few comparable voids also observed at the intragranular twin boundary (Figure 7b). The observation samples were not metallurgically corroded to eliminate interference.

**Figure 8:**
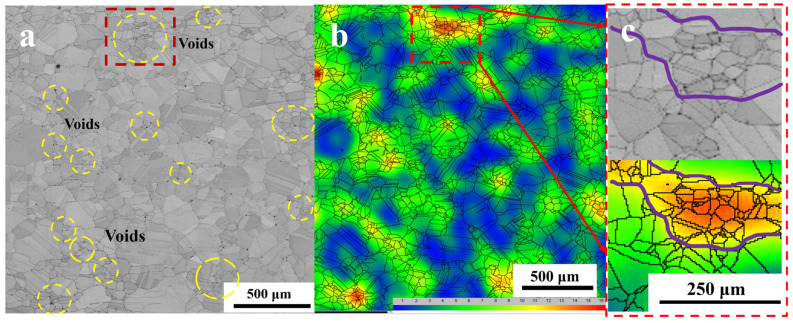
EBSD of electrolytic nickel in atmospheric heat treatment following 8 h at 1100 °C: (**a**) band contrast map; (**b**) strain cloud map; (**c**) local amplification. The red dotted boxes in (**a**,**b**) indicate local areas of high strain. The purple line in (**c**) is also the corresponding high strain region.

Figure 8 displays the EBSD image subsequent to the atmospheric heat treatment. In Figure 8a, the distribution of the grain sizes was non-uniform, where they ranged from several hundred microns for large grains to only ten microns for small grains. Additionally, numerous voids were observed along the grain boundaries, as indicated by the yellow circles in Figure 8a. The strain cloud map depicted in Figure 8b illustrates an uneven strain distribution, with stress levels significantly elevated in proximity to these voids compared with other areas (Figure 8c). The grain size in regions characterized by high-stress values was significantly smaller compared with that of other regions.

#### 3.2.2. Microstructure of Electrolytic Nickel under Vacuum Heat Treatment

The electrolytic nickel was subjected to a vacuum heat treatment to determine whether the formation of pits was related to the external environment.

**Figure 9:**
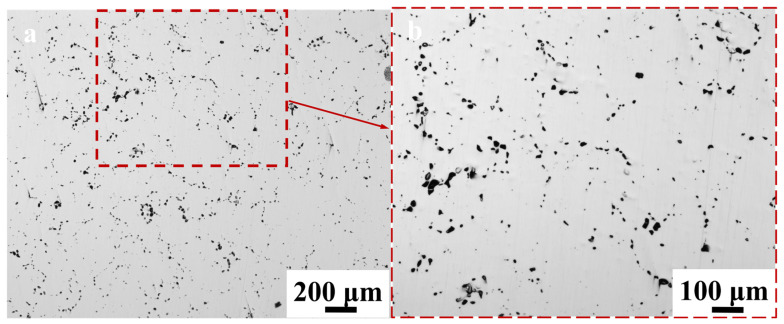
Microstructure of electrolytic nickel heat treatment (non-metallurgical corrosion): (**a**) microstructure following 8 h at 1100 °C in vacuum condition; (**b**) local amplification.

**Figure 10:**
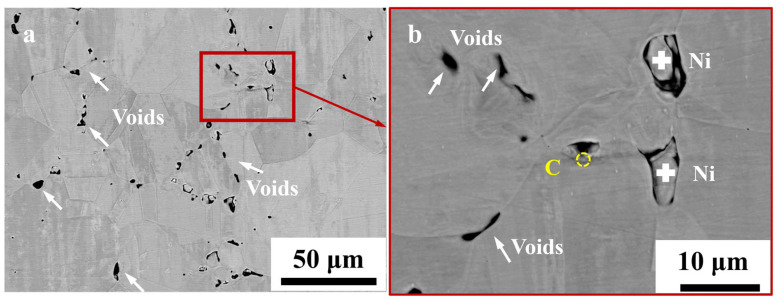
Electron probe microstructure of electrolytic nickel heat treatment (non-metallurgical corrosion): (**a**) microstructure following 8 h at 1100 °C in a vacuum condition; (**b**) voids along grain boundaries; C: Elemental analysis.

The microstructure after the heat treatment at 1100 °C and 8 h in vacuum environment is shown in Figure 9. Similarly, the samples were not metallurgically corroded after grinding and polishing. Affected by elevated temperatures, the grains exhibited substantial growth, which led to the emergence of numerous “black points” along the grain boundaries. These points were distinguished by their backscattered electron contrast (Figure 10). These “black points” represent voids, as shown in Figure 10a. The sizes of the holes varied, where they precipitated along grain boundaries and were unevenly distributed, which aligned with the observations depicted in Figure 7 and Figure 8.

Particles were present in certain perforations, and upon examination with an electron probe, they were identified as nickel. This was debris from the sample grinding and polishing process embedded in the holes. The segregation of carbon was observed exclusively on a limited number of grain boundaries (Figure 10b). During heat treatment under vacuum conditions, it is essential to maintain a very low oxygen partial pressure. In this case, holes still appeared at the grain boundaries. This excluded oxidation by the external environment at high temperatures.

Caplan et al. [20] extensively researched the phenomenon of cavitation and attributed its cause to the presence of carbon within the nickel. During high-temperature oxidation, carbon within nickel creates voids at the grain boundary. Metal cavitation is dependent on the local concentration of carbon segregated at the Ni grain boundaries rather than on the overall carbon content. Additionally, carbon segregation at the nickel grain boundary facilitates the initiation of these voids during grain boundary sliding. When nickel undergoes decarburization prior to oxidation, it prevents the formation of metal cavities. According to Woodford [14], cavitation may occur in nickel when the carbon content is below 100 ppm when exposed to an atmospheric heat treatment. These holes are essentially stomata filled with large amounts of CO gas [18].

The national standard specifies the level of impurity carbon in electrolytic nickel as 0.01%, or 100 ppm. Electrolytic nickel has a critical level of carbon required for cavitation. It is quite possible for cavitation to occur and create holes at grain boundaries.

After being subjected to elevated temperatures in an atmospheric setting, electrolytic nickel undergoes the internal erosion of oxygen atoms along the grain boundaries. These oxygen atoms then encounter carbon atoms diffused to the grain boundaries as impurities, which leads to a reaction that generates carbon monoxide gas, as suggested by previous research [24]. However, after a heat treatment under vacuum conditions, it is unusual for a large number of pores to still appear along the grain boundaries instead of, for example, graphite.

Typically, electroplating solutions are made from electrolyte solutions that contain small amounts of organic and inorganic substances called additives. The co-deposition of additives with reduced metal atoms inevitably occurs, which results in residual impurities in the deposited metal [25,26,27,28]. A possible scenario is that when heated in a high-temperature environment, the composite additives between the electrodeposited layers decompose, which releases a large amount of oxidizing gases [26]. The oxidizing gases erode the grain boundaries and lead to the oxidation of nickel in the deposited layers. Simultaneously, the impurity carbon present in the electrolytic nickel diffuses toward the grain boundary of oxygen-containing nickel at elevated temperatures. Due to the extreme reactivity of the carbon atom, the carbon segregated to the grain boundaries is also oxidized to form CO or CO_2_ gas. After the gas is released, cavitation occurs at the grain boundary, which results in the formation of voids within the grain boundary. This phenomenon can significantly impair the plasticity of the grain boundary. Certain gases persist within the voids of the grain boundaries. When subjected to external forces, these gases can be rapidly released, thus leading to the formation of brittle cracks.

The cavitated grain boundaries cannot migrate properly, and thus, the grains cannot grow. Residual stresses from the electrolytic deposition process are also not released. Higher stresses are retained in these regions and the recrystallization process cannot be completed well. And the co-lattice twin boundaries are not easily oxidized, and thus, it is difficult to observe cavitation at the twin boundaries.

Based on observations, the depth of erosion in the electrolytic nickel during the experiment appeared to be significantly greater than what has been documented in the literature [14,19]. Although the treatment time was not long, cavitation was produced to varying degrees at the grain boundaries of almost all the nickel layers deposited. This indicates that the cause of the holes was in the material itself.

This may explain why a large number of cavitation grain boundaries were still observed during vacuum heat treatment. This was also the reason why a large number of cavitation grain boundaries appeared in this study despite the limited heat treatment time. However, the number of holes that appeared after the vacuum heat treatment was higher than that under atmospheric conditions. This may have been due to the inhomogeneity of the material in the industrial product with a high local concentration of carbon segregation.

### 3.3. Cracking Analysis during Hot-Rolling Process

#### 3.3.1. Macroscopic Defects of Electrolytic Nickel Hot Rolling

**Figure 11:**
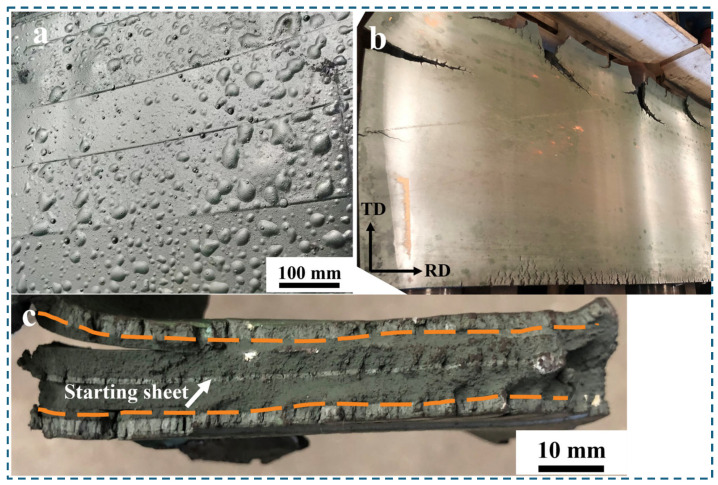
Defects in electrolytic nickel hot-rolling processes: (**a**) surface bulge after heating; (**b**) hot-rolling crack; (**c**) fracture delamination at crack.

The electrolytic nickel was subjected to a heat treatment at 1100 °C in a resistance-heating furnace for a duration of 8 h, which was followed by hot rolling. The hot-rolling process typically occurs at temperatures around 1000 °C. The “bulge” phenomenon (Figure 11a) was noted on a section of the electrolytic nickel plate upon its release, which indicates the outward discharge of gas. The deformation experienced during the initial rolling process was approximately 15%. Subsequently, significant cracks manifested at the periphery of the electrolytic nickel plate in this stage, which rendered further rolling unfeasible (Figure 11b). Observed in the crack fracture was the presence of distinct stratification not only between the starting sheet and the deposited layer but also along the thickness direction of the deposited layer, as illustrated by the dotted line in Figure 11c. The arrows in Figure 11c indicate the nickel starting sheet, which constituted a deposited layer on both surfaces.

#### 3.3.2. Impurities and Residual Gas Elements in Hot-Rolling Cracks

Harmful impurities commonly found in pure nickel and nickel-based superalloys include hydrogen, oxygen, sulfur and phosphorus elements [29,30,31,32,33]. Even when present in small quantities, certain elements can create a eutectic reaction with nickel, which results in a low melting point. Alternatively, they may be situated as a secondary phase at the grain boundaries, which significantly influences the material’s plasticity and high-temperature characteristics. Gas precipitation represents a primary side reaction during the electrodeposition process. This phenomenon frequently diminishes the current efficiency of metal electrodeposition and, in severe instances, can impede the smooth progression of the metal electrodeposition process. The generation of hydrogen during the process of electrodeposition of electrolytic nickel has the potential to cause hydrogen embrittlement [7,34].

The residual carbon, phosphorus, sulfur, oxygen, nitrogen and hydrogen were identified and examined in the cracked sample of the hot-rolled electrolytic nickel, with the results presented in Table 2. The carbon content was 0.01% (100 ppm), while the phosphorus and sulfur contents were 0.001% (10 ppm), all of which fell within the standard range specified in GB/T6516-2010 [35]. The nitrogen content was 5 ppm, which is generally considered harmless. However, a hydrogen content above 2 ppm may lead to hydrogen embrittlement. In this case, the hydrogen content was 0.0004% (4 ppm), which is slightly above the threshold. The oxygen content was significantly higher at 0.0052% (52 ppm). Typically, the oxygen content should not exceed 10 ppm to avoid adverse effects; in this instance, it was fivefold higher.

The concentration of hydrogen was analyzed at 4 ppm based on the elemental content, which could cause embrittlement of the material. However, it did not cause a significant reduction in the overall plasticity of the electrolytic nickel. Nitrogen has a minimal detrimental effect on nickel electrolysis. The presence of the sulfur element in electrolytic nickel typically manifests as a Ni_3_S_2_ phase, which leads to material brittleness. However, after several characterizations, no nickel sulfide phase was found. The levels of sulfur and phosphorus elements in the sample did not surpass the standard limits. And the 10 ppm sulfur content could not have caused such severe cracking. A similar observation was made for phosphorus elements, which also did not contribute to the brittleness.

The oxidation of metals may not be readily apparent at low temperatures; however, once the temperature surpasses a specific critical threshold, the rate of oxidation will markedly escalate. In high-temperature conditions, electrolytic nickel surfaces and fracture surfaces undergo oxidation, which results in the formation of an oxide film on the surfaces. During the entirety of the heating and hot-rolling process, electrolytic nickel undergoes oxidation to varying extents. Figure 11 shows the nickel plate’s surface had undergone oxidation to create an oxide film. Concurrently, the cracked samples also exhibited excessive levels of oxygen content.

Under the effect of the oxygen partial pressure, oxygen atoms in the surrounding environment will progressively diffuse inward along the grain boundary via the outer oxide film. The process of oxygen infiltration persists as the material is thermally exposed to atmospheric conditions. Oxygen atoms fill the vacancies at the grain boundaries, which leads to erosion and weakening of the grain boundaries [14]. Oxygen embrittlement [22,36,37,38], which is a specific type of gas phase embrittlement (GPE), is commonly observed in nickel-based alloys and leads to both static (stress-free) and dynamic (pre-crack propagation) embrittlement.

Bricknell [19] experimentally observed that oxygen undergoes a reaction with the impurity carbon present in nickel, which results in the production of carbon monoxide gases. This reaction leads to the occurrence of grain boundary voids. The formation of grain boundary voids in nickel is noted to occur without the necessity of oxide growth stress or vacancy injection. The presence of carbon significantly influences the formation of grain boundary voids. At elevated temperatures, carbon diffuses toward the grain boundary of nickel-containing oxygen. The compound that interacts with carbon may either be nickel monoxide or atomic oxygen, which is contingent upon the type of oxygen that exists at the nickel grain boundary.

#### 3.3.3. Microstructure of Electrolytic Nickel Hot Rolling

**Figure 12:**
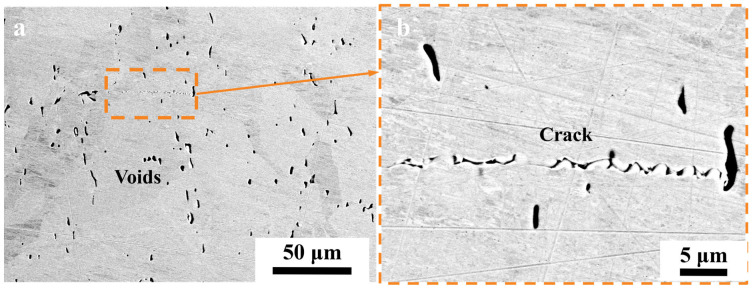
Microstructure in electrolytic nickel after hot rolling (non-metallurgical corrosion): (**a**) distribution of voids in microstructure; (**b**) voids merging along cracks.

**Figure 13:**
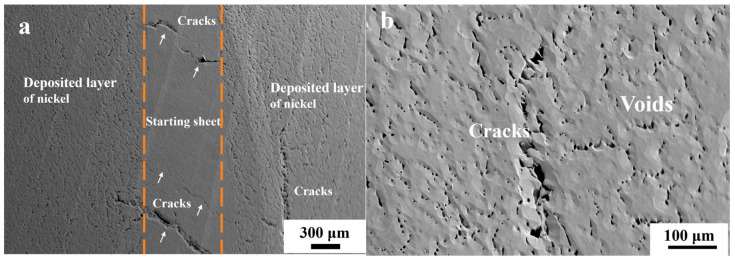
SEM microstructure of crack formation in electrolytic nickel hot rolling: (**a**) cracks in deposited layer and starting sheet; (**b**) cracks in deposited layer.

Figure 12 and Figure 13 depict the microstructure of electrolytic nickel in a hot-rolled state. Figure 12 illustrates the microstructure devoid of metallographic corrosion, which revealed a significant presence of voids within the structure, which aligns with the observations made in Figure 7, Figure 8 and Figure 10. Numerous voids were present at the grain boundary, with a continuous distribution along the boundary; however, only a few voids were detected within the grain.

Under the effect of a rolling force, the apertures depicted in Figure 7 were greatly stretched, which resulted in varying degrees of indentation. Figure 12b illustrates a crack that was present in the structure. The cracks propagated through the voids distributed along the grain boundary under shear stress. It can be inferred that similar cracks were formed through the same process.

Upon examination of the microstructure of electrolytic nickel during hot rolling, it was observed that the microstructural features aligned with the macroscopic cracks present in the hot-rolling process. The material experienced significant plastic degradation, which led to thermal embrittlement.

In Figure 13a, the starting sheet is positioned centrally, bordered by a layer that was deposited. An evident penetrating crack (at the white arrow) was observed on the starting sheet, where it displayed a maximum shear stress distribution at 45 degrees, which suggests that the crack initiation was attributed to shear stress. During the rolling process, the shear slip of the hole-containing grain boundaries resulted in intergranular cracking. The cracks initiated at the interface and propagated toward the interior of the starting sheet. The majority of grain boundaries within the deposited layer structure exhibited extensive cracking, as depicted more prominently in Figure 13b. Significant variations existed in both the quantity and arrangement of perforations noted between the deposited layer and the starting sheet. This was because the impurity elements were more tightly controlled during the deposition process, and the content of impurity carbon elements in the starting electrode sheet was very low. Therefore, the number of pores was small and the weakening effect on the grain boundary was small.

During crystal deformation, the grain boundary experiences shear stress, which leads to the interconnection of pores through this stress and the subsequent formation of cracks [39]. Under the sustained application of the deformation force, the propagated cracks are linked together to create a larger crack. The above conditions in the aforementioned scenario were prevalent in the grain boundaries of this material, which led to severe cracking and a loss of plasticity in electrolytic nickel during the rolling process.

#### 3.3.4. Hot-Rolled Crack Fracture Morphology

**Figure 14:**
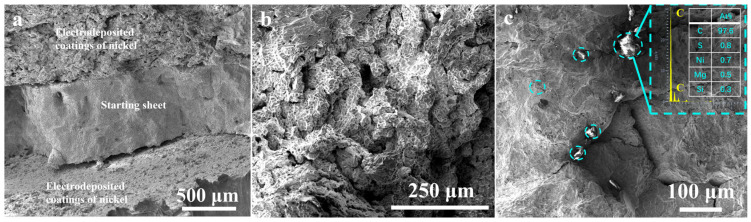
SEM analysis of cracking fracture in electrolytic nickel during hot rolling: (**a**) fracture morphology in starting sheet and deposited layer; (**b**) intergranular cracking in deposited layer; (**c**) intergranular cracking featuring impurities in carbon content. The dotted circles in (**c**) are high carbon areas.

Figure 14 illustrates the fracture morphology at the cracking position of the electrolytic nickel that occurred during the hot rolling. The examination revealed significant cracks between the deposited layer and the starting sheet, which indicates a significant level of material stratification (Figure 14a). Laminar growth is one of the most common forms of electro-crystalline growth of metals. The layer comprises multiple micro-steps, and its formation occurs through the aggregation of these micro-steps. In an industrial electrolyzer, process parameters, such as the nickel ion concentration, temperature and current density, can vary from one deposition layer to another at the interface formed with the electrolyte. Periodic changes in the process parameters or the inclusion of non-metallic substances can lead to the formation of a layered structure [6]. If oxides, inclusions or internal stresses are present at the micro-steps, it indicates a potential weakness in the bonding strength between layers. The layers can be readily separated when exposed to external forces.

The oxidation of electrolytic nickel occurs in a high-temperature setting and is closely linked to the intrinsic properties of the electrolytic material. Oxidizing gases exhibit a higher propensity to infiltrate the matrix from the deposition layer, which leads to erosion of the grain boundary. The presence of oxygen at the grain boundary is associated with the degradation of the grain boundary and a reduction in the intergranular binding strength. Following the reaction between oxygen and carbon, a significant quantity of pores is produced, which leads to a substantial increase in intergranular fracture. This, in turn, results in a deterioration of the processing capabilities of electrolytic nickel, which causes a complete loss of plasticity and renders it incapable of plastic deformation.

The microstructure of the deposited layer appears to be porous (Figure 14b). The porous microstructure resembles the phenomenon known as “overburning” in metal materials. In Figure 14b, the formation of carbon monoxide pores is attributed to the significant erosion of grain boundaries by oxygen. This process involves the combination of carbon with oxygen, which leads to the destruction of intergranular bonds and results in a highly porous microstructure.

In Figure 14c, cracks of various sizes are normally distributed along grain boundaries, which suggests that the propagation paths of the cracks aligned with the intergranular pattern. The energy required for crack propagation along grain boundaries is less than for a through-crystal fracture. After undergoing oxygen erosion at the grain boundary, the crack will persist in propagating along the grain boundary while encountering reduced resistance, which is a phenomenon known as intergranular cracking. In Figure 14c, inclusions were observed at various locations along the grain boundary. The EDS point analysis revealed an enrichment of carbon elements, with a mass percentage that approached 100%. This was carbon segregated at the grain boundaries.

#### 3.3.5. TEM Observation of Electrolytic Nickel Hot Rolling

**Figure 15:**
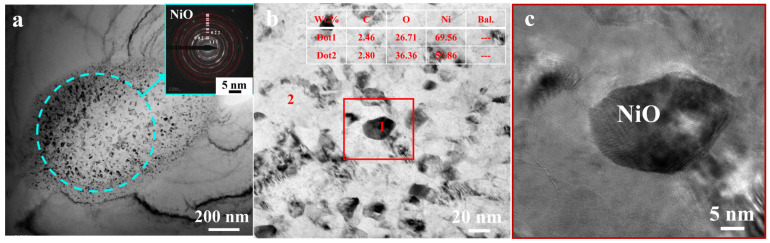
TEM analysis of electrolytic nickel hot rolling: (**a**) aggregates of oxide particles; (**b**) agglomeration of nanoscale oxide particles; (**c**) HRTEM oxide particles.

TEM analysis was conducted on samples of electrolytic nickel following the hot rolling. Inclusion particles were observed at the grain boundaries (Figure 15). The tiny particles were about 1 µm in size and the diffraction pattern was amorphous, which was determined to be NiO after calibration (Figure 15a). The interior of the structure consisted of numerous smaller particles (Figure 15b). High-resolution transmission electron microscopy (HRTEM) analysis was conducted on the region highlighted in the red box (Figure 15c). The analysis of the energy spectrum for points 1 and 2 depicted in Figure 15b indicates that the particulate matter present was nickel oxide. Large quantities of nickel oxide nanoparticles had agglomerated.

Nickel undergoes oxidation easily when heated at 1100 °C atmospheric conditions, such as NiO and Ni_2_O_3_, within the material. Nickel oxides are extremely unstable in high-temperature environments and react with the impurity carbon originally present in electrolytic nickel, and eventually decompose into Ni and CO. The chemical decomposition equation representing the reaction with the lowest energy is depicted in Formula (1). Nickel oxides are generated through thermal decomposition and serve as an additional gas source.

(1) NiO+C→Ni+CO

The potential for oxygen in the atmosphere to engage in reactions with carbon at the grain boundary is subject to regulation by dynamic factors, such as the oxygen partial pressure, temperature and oxygen diffusion rate. Thermal decomposition of the additives that remain between the electrolytic nickel layers increases the oxygen partial pressure and the rate of carbon oxidation. Consequently, this leads to an escalation in the deterioration of the grain boundary and a reduction in the plasticity of the sample. According to the Arrhenius formula (Formula (2)), it is known that the reaction rate constant is

(2) $$k=Aexp\left(-\frac{E_{a}}{RT}\right)$$

where *R* is the gas constant, *A* is the Arrhenius’ constant, *E_a_* is the experimental activation energy and *T* is the thermodynamic temperature. The reaction rate constant *k* exhibits a positive correlation with *T*, which leads to a significant increase in the reaction rate with rising temperatures. The process of heat preservation or hot rolling at a high temperature of 1100 °C facilitates an increase in the rate of reaction between oxygen and carbon, which leads to a rapid acceleration of the reaction at elevated temperatures.

Dyson [24] postulated that the formation of voids on the grain boundaries of nickel would occur at approximately 1000 °C, even in the absence of applied stress. The oxidation reaction between oxygen and carbon at grain boundaries takes place under thermal exposure. The rate of oxygen diffusion is rapid at elevated temperatures, with the diffusion rate at grain boundaries being considerably greater than within the grain. The likelihood of grain boundary diffusion increases in the presence of applied stress. During the process of hot-rolling deformation, there is a higher rate of diffusion and infiltration of oxygen atoms. During grain boundary sliding, cracks initially develop in the highly cavitated grain boundaries, and carbon oxidation persists as the cracks propagate along the grain boundaries. Oxygen embrittlement is attributed to grain boundary pinning induced by oxides or pores, which leads to a hindered grain boundary sliding mechanism rather than an intrinsic decrease in grain boundary binding [22].

In summary, the fracture mechanism of electrolytic nickel hot rolled after a heat treatment at 1100 °C can be proposed as follows.

During the heating and holding process, electrolytic nickel can rapidly develop a compact oxide film on its surface. However, the duration of heating is short, and the availability of oxygen infiltration material in the atmosphere is restricted.

Electrolytic nickel is heated to 1100 °C under atmospheric or vacuum conditions, and the residual additives between the layers of electrolytic nickel are thermally decomposed to release a large amount of oxidizing gases, which subsequently oxidize the grain boundaries. Additionally, impurity carbon atoms present in the electrolytic nickel also diffuse toward the grain boundary. The oxygen and carbon atoms migrate to the unoccupied sites along the grain boundaries and become fixed, which result in the erosion of the grain boundaries. Oxygen encounters impure carbon at the grain boundary, which leads to a reaction that produces gas within the pores. This process results in the formation of voids along the grain boundary, which is known as cavitation. This type of pore is challenging to develop at the twin boundary but forms at an HAGB. Due to the pinning effect of oxygen and carbon atoms on certain grain boundaries, their migration is hindered, which limits the mobility of HAGBs. Consequently, the grain size distribution becomes non-uniform following high-temperature heating. The presence of pinned grain boundaries hinders the sliding motion of grain boundaries, which consequently diminishes the material’s plasticity. The voids experience a high stress concentration, which can lead to easy cracking under force.

As the oxidation reaction advances, the sizes of the voids increase. A significant quantity of cavitation voids is evident along the grain boundary, which significantly impairs the plastic deformation capacity of the grain boundary. Upon the application of the deformation force to the material, these voids will quickly propagate and evolve into numerous cracks, which leads to a significant deterioration of the grain boundary and results in the formation of an intergranular brittle fracture. The grain boundary adjacent to the crack undergoes additional oxidation, which results in an intergranular fracture that is fully coated with oxide.

**Figure 16:**
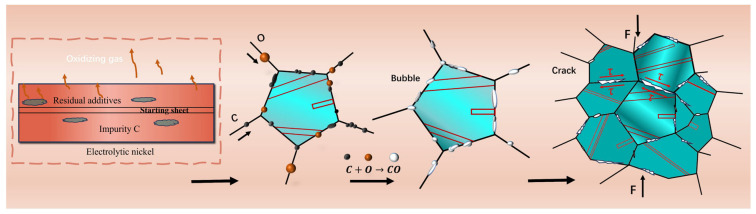
Cracking mechanism in electrolytic nickel hot rolling.

The mechanism that elucidates the reaction between oxygen and carbon, and thus, results in an elevated temperature and reduced plasticity, is postulated based on an examination of industrial electrolytic nickel. In the context of the hot-rolling cracking mechanism of electrolytic nickel, the oxidizing gases, high temperature and specific carbon content are essential factors (Figure 16).

## 4. Conclusions

The mechanism of embrittlement during the hot rolling of industrial electrolytic nickel, which leads to severe cracking during hot rolling, was investigated. Electrolytic nickel exhibits brittle fractures along the grain and very low plasticity during hot rolling. Crack formation is associated with the presence of a large number of holes in the grain boundaries. Whether heat treated under atmospheric or vacuum conditions, holes appear at the grain boundaries. The heat treatment time does not need to be very long for the holes to be distributed throughout the electrolytic nickel material, which suggests that the cause of their formation comes from within the material itself.

Numerous cavitation voids are evident along the grain boundaries, which significantly impairs the material’s plastic deformation capacity. Upon the application of a deformation force (rolling force) to electrolytic nickel, intermittent voids located at the grain boundary penetrate to form cracks due to shear stress. Subsequently, extensive cracking develops along the grain structure. The holes may be caused by the oxidizing atmosphere created by the decomposition of additives left between the electrolytic nickel layers at high temperatures. Oxygen selectively penetrates along the grain boundaries. It then reacts with carbon, which diffuses to the grain boundaries and results in the formation of carbon monoxide gases and intercrystalline voids. This process significantly diminishes the plasticity of the grain boundaries.

The voids manifest on an HAGB, which are pinned by oxygen and carbon atoms, thus making it difficult for migration to occur. This leads to the non-uniformity in the size of recrystallized grains. The scarcity of voids in the twin boundary suggests a low energy level, which makes it challenging for oxygen and carbon to accumulate and bond in this region.

High-temperature conditions, residual additives and carbon are considered to be the direct causes of cracking. It is recommended that the residual additives and carbon content of electrolytic nickel be strictly controlled.

## Figures and Tables

**Table 1:** Main chemical components of electrolytic nickel (wt.%).

Ni	Co	C	Si	P	S	Fe	Cu	Al	Mg
>99.90	0.02410	0.01200	0.02980	<0.0003	<0.0003	0.00819	0.00332	<0.0001	<0.0001

**Table 2:** Elements and gas elements in electrolytic nickel cracking sample (wt.%).

C%	S%	P%	O%	N%	H%
0.01	0.001	0.001	0.0052	0.0005	0.0004

## Data Availability

The original contributions presented in the study are included in the article, further inquiries can be directed to the corresponding author.
